# A Case of a Two-Month-Old Boy Diagnosed with Infantile Crohn's Disease Based on an Atypical Perianal Lesion

**DOI:** 10.1155/2020/8832856

**Published:** 2020-12-09

**Authors:** Kengo Nakaya, Yasushi Iinuma, Yutaka Hirayama, Yu Sugai, Shotaro Taki

**Affiliations:** Department of Pediatric Surgery, Niigata City General Hospital, Shumoku 463-7, Chuo-ku, Niigata 950-1197, Japan

## Abstract

Inflammatory bowel disease is rare in infants, and the early diagnosis is very important. We herein report an infant who received an early diagnosis of infantile Crohn's disease (CD). A two-month-old boy presented with bloody stool. He developed a poor sucking tendency and a painful perianal lesion at three months of age. He was suspected of having infantile CD because of his atypical perianal lesion. Colonoscopy revealed that his perianal lesion had induced rectal longitudinal ulcers. Histology showed no granulomas but patchy inflammation reaching the submucosal layer. He was diagnosed with infantile CD based on the Japanese criteria. CD should be suspected in infants with atypical perianal lesions, irrespective of their age. Early colonoscopy with histology should be considered in these cases in order to prevent adverse outcomes in children.

## 1. Introduction

Inflammatory bowel disease (IBD), primarily a disease of adolescents and young adults, is rare in infants [1, 2]. When it does occur in infants, a delay in the diagnosis and treatment can lead to failure to thrive, so the early diagnosis is very important [3–9].

We herein report an infant who presented with bloody stool and an atypical perianal lesion from two months after birth and received an early diagnosis of infantile Crohn's disease (CD).

## 2. Case Presentation

A boy was delivered at 41 weeks' gestational age with a birth weight of 3210 g. He presented with bloody stool at 62 days old and developed a poor sucking tendency at 3 months old. He showed a painful perianal lesion at 105 days old. He had been managed with a casein-hydrolyzed formula and anti-inflammatory suppository but still had all of his symptoms when he was referred to our hospital at 108 days old.

**Figure 1:**
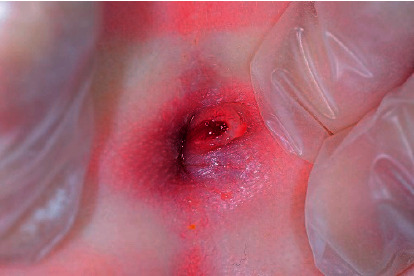
Atypical perianal lesion.

**Figure 2:**
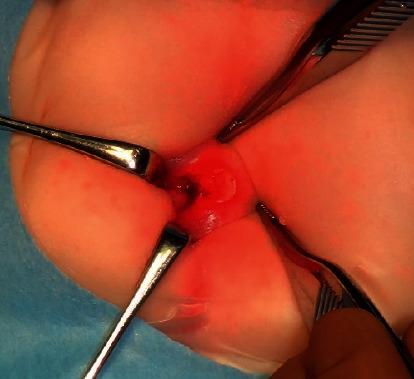
The perianal lesion was confirmed to extend into the anus.

On admission, he was afebrile and active and did not appear septic, and his body weight was 6225 g. A physical examination revealed an atypical perianal lesion with tenderness on palpation (Figure 1). The initial blood screen showed an abnormal hematological profile suggestive of inflammation. His total white blood cell count was elevated at 23.8 × 10^9^/L (neutrophils 63.4%, lymphocytes 22.3%, and eosinophils 6.0%). The C-reactive protein level was elevated at 2.69 mg/dl. At this time, he was suspected of having infantile CD because of his atypical perianal lesion. Two days after admission, a transanal rectal biopsy was performed under general anesthesia, and the specimen showed nonspecific rectal inflammation with no definitive findings of ulcerative colitis (UC) or CD. Under general anesthesia, the perianal lesion was confirmed to extend into the anus (Figure 2), inducing rectal ulcer with a diameter of about 1.5 cm.

**Figure 3:**
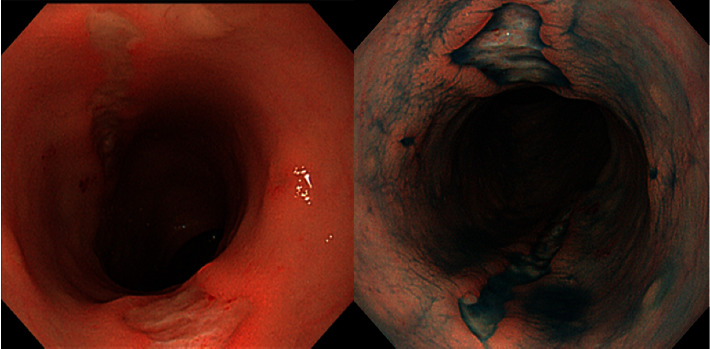
Colonoscopy showed rectal longitudinal ulcers.

**Figure 4:**
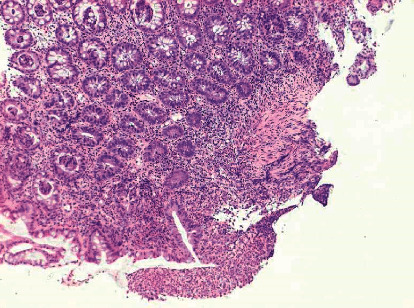
Histology showed no granulomas but patchy inflammation reaching the submucosal layer (10x magnification).

Use of an elemental diet improved his bloody stool, his sucking tendency, and his blood examination findings but did not improve the perianal lesion with tenderness on palpation. At 117 days old, colonoscopy showed two rectal longitudinal ulcers (Figure 3). Histology showed no granulomas but patchy inflammation reaching the submucosal layer (Figure 4). He was strongly suspected of having infantile CD and transferred to another hospital in order to receive treatment at 123 days old.

## 3. Discussion

The onset of IBD of infancy is very rare, and the onset of CD in infancy accounts for only 1% of total pediatric CD cases [1, 2, 7]. It is difficult to make an early diagnosis of IBD because similar symptoms are seen in enterocolitis, which is common in infants, as well as food protein-induced enterocolitis syndrome (FPIES), the incidence of which has been on the rise [10]. Gryboski et al. reported that the average duration of symptoms before the diagnosis was 6 ± 2.6 months [11]. Furthermore, it is known that an early differential diagnosis of CD and UC is difficult in early-onset IBD. Benchimol reported that the likelihood of the diagnosis changing from UC to CD was about 12% and from CD to UC was about 3% [12]. In addition, there is a disease concept of indeterminate colitis (IC) for patients with features of both CD and UC [13]. However, we believe that it is important to at least suspect IBD (not to make a detailed differential diagnosis) and perform endoscopy early.

Table 1 reviews the literature concerning infantile CD, including our case [3–9]. There have been 20 cases, and half of them required about 6 months to diagnose. The median duration of symptoms before the diagnosis was 6.5 months (range 0–27 months), which was longer than the duration reported by Gryboski and Greef in their studies of CD in children [11, 14]. The diagnosis of infantile CD therefore seems to be more difficult than that of CD in children. A perianal lesion was confirmed in 13 cases (65%), which seems to be a higher incidence than in previous reports on CD in children [14–16]. A persistent perianal lesion is therefore an important finding in cases of infantile CD. Indeed, we strongly suspected CD in the present case after examining his atypical anal lesion and noting findings similar to those of the case 3 patient, who had been treated at a related institution [5].

The present patient consulted a doctor after 28 days from his first symptoms were noted, and the diagnosis of CD was made based on colonoscopy performed 10 days after being admitted to our institution. Because the histology of a transanal rectal biopsy showed no granulomas or specific rectal inflammation with definitive findings of IBD, a very early diagnosis was not able to be made. Early colonoscopy with histology instead of the first transanal rectal biopsy could probably made the diagnosis of CD a week earlier.

## 4. Conclusions

CD should be suspected in infants with atypical perianal lesions, regardless of their age. In the absence of classic features, the diagnosis of CD can be difficult and delayed, as has been noted in previous cases, so early colonoscopy with histology should be considered in these cases in order to avoid adverse outcomes for children.

## Figures and Tables

**Table 1:** A review of the literature concerning infantile CD, including our case.

No	Author, year	Age at onset	Age at diagnosis	Symptoms	Perianal lesion
1	Mezoff AG, 1990	4 weeks	11 months	Vomiting, bloody diarrhea, perianal inflammation with anal fissure	+
2	Bryk T, 1995	9 months	9 months	Bloody diarrhea, severe perianal inflammation with anal fissures	+
3	Yagi M, 1995	6 months	23 months	Fever, bloody diarrhea, anal fissure	+
4	Pashankar D, 2000	3 months	15 months	Fissure, abscess, multiple fistulae in the perianal area	+
5		9 months	3 years	Dyschezia, bloody stools, perianal skin tags	+
6		9 months	2 years	Multiple perianal fistulae after perianal abscess	+
7	Cohen Z, 2000	NA	2 weeks	FTT, bloody diarrhea, severe perianal disease	+
8		NA	3 weeks		+
9		NA	2 months		+
10		NA	3 months		+
11	Harboe ZB, 2001	3 months	5 months	Diarrhea, fever, FTT, anemia, inflammation of the oral mucosa.	−
12	Marx G, 2002	1 month	12 months	Bloody diarrhea	−
13		2 months	21 months	FTT, bloody diarrhea	−
14		5 months	10 months	FTT, bloody stools	−
15		9 weeks	11 weeks	FTT, bloody diarrhea	−
16		5 weeks	7 months	Dehydration, intractable diarrhea	−
17		1 month	8 months	FTT, chronic diarrhea	−
18	Shim JO, 2013	1 month	8 months	FTT, bloody diarrhea, skin folliculitis	+
19		3 days	4 months	Bloody diarrhea, pus discharge from labia majora	+
20	Our case	2 months	3 months	Bloody stools, poor sucking, untypical perianal lesion	+

FTT: failure to thrive; NA: not available.
